# Treatment of Chronic Pulmonary Heart Disease with Traditional Chinese Medicine: A Protocol for the Development of a Core Outcome Set (COS-TCM-CPHD)

**DOI:** 10.1155/2021/5559883

**Published:** 2021-04-12

**Authors:** Bohan Niu, Mingyan Zhang, Hui Zi Chua, Kai Li, Junhua Zhang

**Affiliations:** ^1^Evidence-Based Medicine Centre, Tianjin University of Traditional Chinese Medicine, Tianjin, China; ^2^Chinese Clinical Trials Core Outcome Set Research Centre, Tianjin, China

## Abstract

**Background:**

Treatment of chronic pulmonary heart disease (CPHD), a common disease, has over recent years been studied using traditional Chinese medicine (TCM) due to many high-profile benefits. These can be evaluated by the measurement and analysis of related outcomes. Because of selective reporting bias and the heterogeneity of study outcomes, it is not possible to combine similar studies in a meta-analysis. Consequently, not only does the low quality of original studies fails to support evidence-based decision-making, but also the value of those clinical studies cannot be evaluated. To solve these problems, the development of a core outcome set for traditional Chinese medicines for the treatment of chronic pulmonary heart disease (COS-TCM-CPHD) is required.

**Methods:**

The development is conducted in five steps: (1) a library of outcomes through systematic review, the retrieval of libraries from two clinical trials registries, and semistructured interviews is established; (2) following data extraction and analysis of the library of outcomes, each outcome can be classified into seven outcome domains, including TCM disease, symptoms/signs, physical and chemical testing, quality of life, long-term prognosis, economic evaluation, and adverse events to form a preliminary list of outcomes; (3) stakeholder groups for participation are selected; (4) stakeholder groups are invited to participate in two rounds of Delphi surveys to score outcomes and provide additional outcomes; (5) a consensus meeting is organized to produce the final COS-TCM-CPHD. *Discussion.* The protocol is consistent with the guidelines defined by the Core Outcome Set-STAndardised Protocol (COS-STAP) statement and formulated with reference to Core Outcome Set-STAndards for development (COS-STAD). The COS-TCM-CPHD will improve the consistency of study reports and reduce publication bias, thereby improving the quality of TCM clinical trials and decision-making for evidence-based medicine. The study has been registered on the COMET website (http://www.comet-initiative.org/Studies/Details/1677).

## 1. Introduction

Chronic pulmonary heart disease (CPHD) comprises a number of disorders (such as those of the bronchus, chest wall, and circulatory system) that raise the pressure in the pulmonary artery and modify the structure and function of the right ventricle [1, 2]. The clinical manifestations include cough and asthma, shortness of breath, palpitation, cyanosis, and edema. A recent study indicated that the pathological mechanisms of CPHD principally involve a state of chronic hypoxia, secretion of vascular mediators such as nitric oxide and endostatin-1 (ET1), and a reduction in platelet-derived growth factors (PDGFs) A and B, leading to the relaxation of and damage to vascular smooth muscle cells, eventually causing an increase in pulmonary arterial pressure (mean value >20 mmHg) [3–6]. CPHD accounts for 6% to 7% of all types of heart disease in adults. The incidence of CPHD varies greatly from country to country and depends on air pollution, rates of smoking, and a variety of other risk factors for lung disease [1].

Although many studies of treatments for CPHD have been published, no effective treatment has been developed to reduce the rate of mortality or disability caused by CPHD. It causes a huge economic burden on both the patient's family and society. The purpose of treatment is to improve oxygenation and right ventricular function. Conventional treatment strategies include increasing right ventricular contractility and reducing pulmonary hypertension while treating the underlying disease. Conventional treatments include cough and asthma drugs, expectorants, vasodilators, inotropic agents, and diuretics, which effectively improve the clinical symptoms [2]. However, conventional treatments cannot reduce the recurrence of symptoms, the patient's quality of life remains poor, and the course of the disease does not become substantially shorter [7]. Although insufficient evidence exists to demonstrate that the use of conventional drugs adversely affects patient outcomes, long-term use can cause arrhythmia, systemic hypotension, acid-base balance disorders, and other toxic side-effects [8, 9].

In clinical trials, issues such as selective reporting of outcomes, high heterogeneity of outcomes, and an inability of research results to be replicated in relevant subjects may cause difficulties when attempting to combine the results with similar research studies, while studies often do not truly reflect the requirements of participants and waste limited research resources [10–12]. A recent systematic review evaluating ginkgo biloba extract and injections of dipyridamole for the treatment of CPHD included 28 studies with 2,457 patients. The study concluded that many key outcomes closely related to patient survival were not reported, preventing conclusions from being drawn on important outcomes [13]. A systematic review of digoxin treatment for CPHD by Alajaji et al. pointed out that the included outcomes were very heterogeneous; thus, it was not possible for similar studies to be combined for analysis [14]. In a systematic review of 35 clinical trials with 2,715 patients that evaluated Danshen by injection for the treatment of CPHD, the majority of the studies reported only laboratory outcomes, while the important clinical outcomes were not reported. Thus, only positive outcomes were reported and otherwise reported selectively, likely leading to inappropriate or even incorrect conclusions [15].

Over recent years, an increasing number of clinical studies of treatments for chronic cor pulmonale using traditional Chinese medicine (TCM) have been published, the efficacy of which has gradually become more well-known. Treatments for CPHD using TCM have been shown to be anti-inflammatory, prevent platelet aggregation, strengthen heart function, and improve immunity and microcirculation [13, 16, 17]. However, clinical trials of TCM should measure and analyze relevant outcomes. Recently, we have found a number of problems when analyzing the outcomes of treatments for CPHD: ① clinical studies of the same condition studied different outcomes, different numbers of outcomes, and variable outcome measurement time points; such problems lead to bias in the reporting of results. ② The choice of outcomes was arbitrary, lacking any uniformity in standards. ③ The results did not correlate with outcomes that patients care about; for example, studies rarely described outcomes such as quality of life or long-term prognosis for patients instead of describing physical and chemical testing and other similar outcomes. ④ The outcomes lacked uniformity in the standards related to TCM. Low-quality original research not only fails to provide the evidence required for evidence-based decision-making but also fails to reflect the potential value of each clinical study. Thus, the correct selection of outcomes is of utmost importance in clinical research [18].

A core outcome set (COS) is a description of the least and most important outcomes, which should be measured and reported in all clinical trials in the same health field [19]. The development of a core outcome set for traditional Chinese medicine in chronic pulmonary heart disease (COS-TCM-CPHD) would minimize the heterogeneity of reported results and improve the methodological quality of clinical research in TCM, important for international recognition.

## 2. Methods/Design

This study aimed to develop a set of COS based on a series of standards [20–23] formulated by the Core Outcome Measures in Effectiveness Trials (COMET) group for future clinical research of CPHD in TCM. The study has been registered on the COMET website, with registration number 1677 [24].

## 3. Study Design

**Figure 1:**
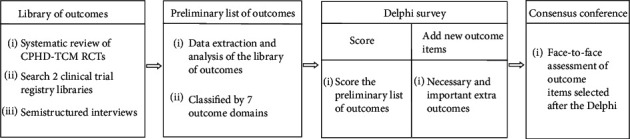
Flowchart illustrating the development of the COS-TCM-CPHD.

The development of the COS-TCM-CPHD is conducted using a five-step process (Figure 1).

Stage 1: establish a library of outcomes through systematic review, retrieval of the libraries from clinical trials registries, and semistructured interviews. Stage 2: after data extraction and analysis of the library of outcomes, the data will be classified into seven outcome domains, including TCM disease, symptoms/signs, physical and chemical testing, quality of life, long-term prognosis, economic evaluation, and adverse events to form a preliminary list of outcomes. Stage 3: stakeholder groups are selected for participation. Stage 4: stakeholder groups are invited to participate in two rounds of Delphi surveys to score the outcomes and establish additional outcomes. Stage 5: a consensus meeting is convened to produce the final COS-TCM-CPHD.

## 4. Steering Committee

The steering committee includes clinical experts in cardiovascular medicine, respiratory medicine, and TCM, a methodological expert, a clinical researcher, a policymaker, and a COS developer. The function of the steering committee is to review and provide guidance at each stage of the research program.

## 5. Working Group Constitution

A working group consisting of 15 individuals, including ten professionals and five representatives of CPHD patients, is constituted. The role of the working group includes the distribution of questionnaires, organization of regular meetings, facilitating communication, holding discussion meetings, and soliciting opinions from the steering committee when any differences require resolution.

## 6. Patient and Public Involvement (PPI)

In order to protect patients' rights to informed consent, a function of the working group is to fully communicate with CPHD patients, such as informing patients of the relevant concepts, the background, purpose, and research methods of this research study. The voluntary participation of CPHD patients or their representatives in semistructured interviews, two rounds of Delphi surveys, and the final consensus meeting is anticipated.

### 6.1. Stage 1: Establishment of a Library of Outcomes

#### 6.1.1. Systematic Review of Randomized Controlled Trials (RCTs) and Systematic Reviews of RCTs for TCM

*(1) Literature Search*. Collection of evidence was conducted independently by two researchers through a search of a total of seven electronic databases, namely PubMed, Cochrane Library, Embase (3 English databases), Chinese Biological Medicine Database, China National Knowledge Infrastructure, Wanfang Database, and the Chinese Scientific Journal Database (4 Chinese databases). The key terms for the search include ‘pulmonary heart disease', ‘cor pulmonale', ‘pulmonary heart diseases', ‘TCM', ‘herbal medicine', and ‘Chinese herbal drugs'. Two researchers will independently conduct the literature search. Any differences will be resolved through discussion or consultation with a third researcher. The inclusion and exclusion criteria of this study are displayed in Table 1.

*(2) Data Extraction*. Data extraction was conducted independently by the two researchers, recording the following information: name of the first author, sample size, basic characteristics of the included subjects (such as age and gender), type of TCM syndrome, intervention details (drug name, course of treatment, treatment frequency, and dose), and outcomes (name or definition, measurement method, and measurement time point).

#### 6.1.2. Search of the Libraries of Two Clinical Trial Registries

A search of clinicaltrials.gov [25] and the Chinese clinical trials register [26] independently by two researchers of all clinical trial protocols for CPHD was conducted for interventions using TCM.

*(1) Data Extraction*. The data for extraction include the country where the registered organizations/researchers are located, the status of the registered trials, ethics, sources of funding, stage of research, details of the intervention, description of outcomes, method of outcome measurement, and time points of the measured outcomes.

#### 6.1.3. Semistructured Interviews

*(1) Interviewee*. Interviewees are selected after obtaining fully informed consent, representing professional doctors in the cardiovascular or respiratory field and patients or caregivers who are able to communicate freely and with a strong capability for comprehension.

*(2) Hospital Selection*. To fully incorporate the views of clinicians, five hospitals (including outpatient and inpatient departments) of integrated traditional Chinese and Western medicine at different levels (levels one, two, and three) across a variety of regions are selected.

*(3) Interview Outline Design*. Due to the particularities of different groups of doctors and patients, outlines of the interviews for doctors and patients required separate designs.

The open communication method was used in the interviews with doctors. By considering doctors as the main body, they can freely list the outcomes they consider important, thereby avoiding restrictions of outcome selection.

A combination of guided and open communication methods was used in the interviews with patients. After the working group has introduced the concepts and research goals of the study to CPHD patients in easy-to-understand terms, interviewers aimed to converse through inquiry and guidance, using the following questions: “What do you care about the most about this disease?”, “What changes do you want in your body?”, and “What level of rehabilitation do you hope to achieve?” The full expression of results that patients consider important is permitted.

*(4) Interview Format*. For professional doctors in the cardiovascular or respiratory fields, communication is via mobile phone video or face-to-face interviews. As patients do not have professional knowledge, communication with CPHD patients is via face-to-face interviews.

### 6.2. Stage 2: Compilation of a Preliminary List of Outcomes

#### 6.2.1. Data Analysis

The extracted outcomes are imported into an Excel spreadsheet for sorting; the outcomes were matched with original research to facilitate the identification of the source, performed independently by two researchers, with differences resolved through discussion or consultation with a third researcher until a consensus is reached.

The process comprises three steps:

1. Deduplicate outcomes and record all the research numbers and quantities that report the outcomes and the frequency of use of each outcome.
2. Standardize the extracted original outcomes to unify and standardize the names, specifically to include abbreviations, nicknames, splits, and mergers. The standardization process is conducted in a manner that ensures the original intent remains unchanged, and the same outcomes are merged and classified.
3. Organize and record the description and frequency of all outcomes to form an initial list of outcome items.

#### 6.2.2. Outcome Domains

From the functional attributes of the outcomes, it is planned to categorize the initial outcome items by reference to seven outcome domains: TCM disease, symptoms/signs, physical and chemical testing, quality of life, long-term prognosis, economic evaluation, and adverse events.

#### 6.2.3. Determination of a Preliminary List of Outcomes

The number of outcome items directly affects the efficacy of the response [20]. If the number of outcomes is small, all are included in the preliminary list of outcomes; if there are more than 80 outcomes, specific criteria can be applied to shorten the preliminary list. For example, the steering committee is asked to remove from the preliminary list of outcomes any item for which at least 80% of members have voted in favor of removing it by internal voting. During the process, the working group can also list other important outcomes not in the preliminary list. Finally, approval of the reserved outcomes and additional outcomes by the steering committee can serve as the preliminary list of outcomes.

### 6.3. Stage 3: Selection of Stakeholder Groups for Participation

The selection of stakeholder groups is crucial for the cost-effective development process [27–29]. The study intends to select five stakeholder groups, including COS users, cardiovascular and respiratory medical professionals (experts in Chinese medicine and clinical experts of Western medicine), patient representatives, methodologists, and health policymakers. Representatives of stakeholder groups can participate in subsequent Delphi surveys and consensus meetings.

### 6.4. Stage 4: Delphi Survey

A Delphi survey is an electronic questionnaire. An email was sent to each stakeholder through the online Delphi survey software containing a link to the electronic questionnaire in which participants score outcome items; the collected scores are used for statistical analysis.

Scoring is based on methods used in previous COS studies, using 1–9 points and “undefined.” Scores of 1–3 are categorized as “not important,” 4–6 as “important but not critical,” and 7–9 as “critical”; if participants are not sure whether the outcome is important, “uncertain” can be selected [30].

At present, there is no international consensus on the optimal sample size for participation in Delphi surveys. The larger the sample size of stakeholders, the better the results. For this study specifically, the goal is to recruit 150 stakeholders, a total of 30 individuals in each interest group.

Participants in the second round of the Delphi surveys are those who completed the first round. If no response is received between rounds, it is inevitable that fewer and fewer members will eventually complete the Delphi survey, leading to attrition bias. Reminder emails are sent after each round of surveying to reduce follow-up bias. Through voluntary participation, the purpose and importance of the research should encourage, as far as possible, the completion of the questionnaire.

After starting the electronic questionnaire, the data are not submitted if the participants give up partway through. Therefore, there are only two possible outcomes: completion of the questionnaire or nonparticipation, ensuring there are no missing data.

#### 6.4.1. Round 1

*(1) Implementation Process*. In the first round of implementation, an email was sent to participants other than patients through the Delphi survey software, containing an invitation letter and a link to the questionnaire. After participants open the link and complete the registration with basic information such as name, email, and phone number, data such as research topics and outcome items will be displayed. The scoring of each outcome item is based on the scoring criteria described above. Considering that some representatives of CPHD patients may not use email, paper questionnaires can be distributed by the working group. For scoring, CPHD patient representatives are required to complete the survey under the supervision of a professional doctor or trained working group member to better understand and ensure the scoring accuracy. Additionally, participants can contribute additional outcome items they think necessary for COS-TCM-CPHD via an open-ended question at the end of the questionnaire: “Do you think there are any important additional indicators that need to be added?” It is planned that this round of investigation is completed within four weeks.

*(2) Data Statistics and Analysis*. After completion of the first round of scoring, statistical analysis can be conducted using the automatic descriptive analysis function of the Delphi survey software, in which the distribution of each item's score is recorded and presented visually. For each question, the number of participants and number of responses ultimately establish whether items are retained, namely, those results defined as “critical” in at least one stakeholder group (≧70% of participants scoring the result as 7–9 points or ≦20% of the participants scoring the result 1 to 3 points), from the consensus definitions described in Table 2. Retention or disposal of all other outcomes is accomplished via a discussion by the working group, as appropriate.

All retained outcomes are included in the second round of the Delphi survey.

#### 6.4.2. Round 2

After completion of the first round of the Delphi survey, it is intended that stakeholders who participated in the first round complete the second round of the Delphi survey.

*(1) Feedback*. In the second round, the following information is made available to all participants: (1) outcome items additional to the first round; (2) number of outcome items and a summary of the distribution of scores from all participants; (3) participant's own score for each indicator in the first round.

*(2) Implementation Process*. The aim of providing this feedback is that participants rescore each item using the scoring criteria described above. If a participant's score changes significantly from the first round, the reason for the change must be explained. Additional and important outcome items may still be added in this round, which should be completed within four weeks.

*(3) Data Statistics and Analysis*. After the second round, the working group recorded the number of participants and respondents. From the percentage of points scored by each stakeholder group after the second round and the consensus definition in Table 2, items that reached consensus in at least one stakeholder group (at least 70% of the participants rating an outcome as 7–9 points or ≦20% of the participants scoring the outcome 1 to 3 points) must be included in the COS-TCM-CPHD candidate outcome items. Any remaining items can also be discussed in the consensus meeting.

*(4) Definition of Consensus*. Each consensus is divided into three categories: “consensus in,” “consensus out,” or “no consensus.” If at least 70% of the participants rated an outcome as 7 to 9 or ≦20% of participants rated the outcome as 1 to 3, the item is prioritized for inclusion in the candidate list of core outcomes, defined as “consensus in.” When more than 75% of the participants rated an outcome from 1 to 3 points or ≦10% rate an outcome as 7–9 points, it is defined as “consensus out.” “No consensus” refers to outcomes that do not fulfill either of the definitions above (Table 2) [30].

### 6.5. Stage 5: Consensus Meeting

A one-day face-to-face national meeting in Tianjin, China, of approximately 20 people (at least 4 for each stakeholder) is anticipated. An online video conference can be scheduled if special circumstances arise (such as a recurrence of COVID-19). Participants should mainly be senior representatives of stakeholder groups and experts in related fields who have completed the two rounds of Delphi surveys.

The following is anticipated:

1. Representatives of the working groups report the Delphi investigation process and the COS candidate items to the conference experts.
2. After full discussion, representatives from different stakeholder groups vote anonymously.
3. If ≧70% of the participants rate an outcome 7–9 points or ≦20% rate an outcome from 1 to 3, that outcome be recommended first [31].
4. All “no consensus” outcomes should be discussed again to reach a consensus. If there is a conflict of opinion, a nominal group method can be adopted to resolve the situation.

The consensus meeting was designed such that the working group can establish the final COS-TCM-CPHD.

## 7. The COS-TCM-CPHD Report

Ultimately, it is intended that the COS-TCM-CPHD be described in accordance with the COS-STAR [23] to ensure transparency and completeness of the report. In a subsequent promotion and application process, the COS-TCM-CPHD should be regularly evaluated to ensure that it is practical and is continually refined.

## 8. Discussion

This study protocol is the first COS-TCM-CPHD to be registered on the COMET website. As a complementary and alternative medicine, TCM plays an important role in the healthcare system [32]. The use of COS in the synthesis and conversion of evidence is essential [12]. As increasing numbers of clinical trials using TCM are undertaken, the development of COS-TCM provides the standardization of TCM clinical trial results, improving the quality of evidence for TCM.

The research plan of the COS-TCM-CPHD strictly follows the best method guidelines provided by the COMET initiative (COS-STAD minimum standard [22] and COS-STAP statement [21]), learning from COS protocols that have been published successfully in other fields.

The protocol design establishes a comprehensive review of the international and Chinese literature by conducting semistructured interviews, Delphi surveys, and a consensus meeting to fully adopt the views of multiple stakeholder groups to ensure the feasibility and promotion of COS-TCM-CPHD in future clinical studies. The development of COS-TCM-CPHD ensures the consistency of reporting clinical study outcomes for TCM in the future, assisting in reducing reporting bias. The results of different clinical trials can be compared and merged in the future to improve the value of clinical studies and reduce the waste of study resources. It is hoped that the development of COS-TCM-CPHD can improve the methodological quality of TCM-related clinical trials and meet international standards.

## Figures and Tables

**Table 1:** Inclusion and exclusion criteria.

Inclusion criteria	Exclusion criteria
1. P : patients with a clear diagnosis of CPHD	1. Studies of acute exacerbation of CPHD
2. I : intervention measures including TCM treatment	2. Duplicate publications or literature comprising only an abstract
3. O : outcomes included in all studies	3. Studies where the complete raw data cannot be obtained
4. S : RCTs and systematic review of RCTs	4. Literature not written in either Chinese or English
	5. Comorbidities and complications
	6. Animal studies

**Table 2:** Definition of consensus.

Consensus in	At least 70% of the participants rated an outcome as 7 to 9 or ≦20% of the participants rated the outcome as 1 to 3
Consensus out	≧75% of the participants rated an outcome as 1 to 3 points or ≦10% of the participants rated the outcome as 7–9 points
No consensus	Anything else

## Data Availability

The data that support the findings of this study are available from the corresponding author upon reasonable request.

## Abbreviations

CPHD: Chronic pulmonary heart disease
COS: Core outcome set
TCM: Traditional Chinese Medicine
COS-TCM: Core outcome set of traditional Chinese medicine
COS-TCM-CPHD: Core outcome set of traditional Chinese medicine for chronic pulmonary heart disease
PPI: Patient and public involvement
COMET: Core outcome measures in effectiveness trials
COS-STAP: Core Outcome Set-STAndardised Protocol
COS-STAD: Core Outcome Set-STAandards for Development
COS-STAR: Core Outcome set-standards
PPI: Patient and public involvement.

## References

[1] Garrison D. M., Pendela V. S., Memon J. (2020). Cor pulmonale. *StarPearls*.

[2] NICE Guideline Updates Team (UK) (2018). *Managing Pulmonary Hypertension and Cor Pulmonale: Chronic Obstructive Pulmonary Disease in over 16s: Diagnosis and Management*.

[3] Lee S. (2019). Comprehensive nursing management for valvular disease. *Critical Care Nursing Clinics of North America*.

[4] Yoon Y. S., Jin M., Sin D. D. (2019). Accelerated lung aging and chronic obstructive pulmonary disease. *Expert Review of Respiratory Medicine*.

[5] Smolders V. F., Zodda E., Quax P. H. A. (2018). Metabolic alterations in cardiopulmonary vascular dysfunction. *Frontiers in Molecular Biosciences*.

[6] Sakao S. (2019). Chronic obstructive pulmonary disease and the early stage of cor pulmonale: a perspective in treatment with pulmonary arterial hypertension-approved drugs. *Respiratory Investigation*.

[7] Kim M., Tillis W., Patel P., Davis R. M., Asche C. V. (2019). Association between asthma/chronic obstructive pulmonary disease overlap syndrome and healthcare utilization among the US adult population. *Curr Med Res Opin*.

[8] Denault A. Y., Bussières J. S., Arellano R. (2016). A multicentre randomized-controlled trial of inhaled milrinone in high-risk cardiac surgical patients. *Canadian Journal of Anesthesia/Journal Canadien D’anesthésie*.

[9] Herman L. L., Bashir K. (2020). *Hydrochlorothiazide*.

[10] Chalmers I., Glasziou P. (2009). Avoidable waste in the production and reporting of research evidence. *Obstetrics & Gynecology*.

[11] Hutton J., Williamson P. (2000). Bias in meta-analysis with variable selection within studies. *Journal of the Royal Statistical Society Series C*.

[12] Chan A. W., Song F., Vickers A. (2014). Increasing value and reducing waste: addressing inaccessible research. *Lancet*.

[13] Qiu J., Guo Y., Xu X., Yue H., Yang Y. (2020). Ginkgo leaf extract and dipyridamole injection for chronic cor pulmonale: a PRISMA-compliant meta-analysis of randomized controlled trials. *Bioscience Reports*.

[14] Alajaji W., Baydoun A., Al-Kindi S. G., Henry M. S. L. S. L., Hanna M. A., Oliveira G. H. (2016). Digoxin therapy for cor pulmonale: a systematic review. *International Journal of Cardiology*.

[15] Liu Y., Huang Y., Zhao C. (2014). Salvia miltiorrhiza injection on pulmonary heart disease: a systematic review and meta-analysis. *The American Journal of Chinese Medicine*.

[16] Shi L., Xie Y., Liao X., Chai Y., Luo Y. (2015). Shenmai injection as an adjuvant treatment for chronic cor pulmonale heart failure: a systematic review and meta-analysis of randomized controlled trials. *BMC Complementary and Alternative Medicine*.

[17] Lu Y., Jin W., Zhang H., Xiaoyun Z. (2016). [Multicenter clinical efficacy observation of integrated Traditional Chinese Medicine-Western medicine treatment in acute onset period of pulmonary heart disease]. *Journal of Traditional Chinese Medicine*.

[18] Glasziou P. (2014). The role of open access in reducing waste in medical research. *PLoS Medicine*.

[19] Williamson P., Altman D., Blazeby J., Clarke M., Gargon E. (2012). Driving up the quality and relevance of research through the use of agreed core outcomes. *Journal of Health Services Research & Policy*.

[20] Williamson P. R., Altman D. G., Bagley H. (2017). The COMET handbook: version 1.0. *Trials*.

[21] Kirkham J. J., Gorst S., Altman D. G. (2019). Core outcome set-STAndardised protocol items: the COS-STAP statement. *Trials*.

[22] Kirkham J. J., Davis K., Altman D. G. (2017). Core outcome set-STAndards for development: the COS-STAD recommendations. *PLoS Medicine*.

[23] Kirkham J. J., Gorst S., Altman D. G. (2016). Core outcome set-STAndards for reporting: the COS-STAR statement. *PLoS Medicine*.

[24] Zhang J. H., Zhang M. Y., Niu B. H. (2020). Developing a core outcome set of traditional chinese medicine on chronic pulmonary heart disease. http://www.comet-initiative.org/Studies/Details/1677.

[25] https://www.clinicaltrials.gov/

[26] http://www.chictr.org.cn/

[27] Dawson J., Doll H., Fitzpatrick R., Jenkinson C., Carr A. J. (2010). The routine use of patient reported outcome measures in healthcare settings. *BMJ*.

[28] Arnold L. M., Crofford L. J., Mease P. J. (2008). Patient perspectives on the impact of fibromyalgia. *Patient Education and Counseling*.

[29] Klokker L., Osborne R., Wæhrens E. E. (2015). The concept of physical limitations in knee osteoarthritis: as viewed by patients and health professionals. *Quality of Life Research*.

[30] Sinha I. P., Smyth R. L., Williamson P. R. (2011). Using the Delphi technique to determine which outcomes to measure in clinical trials: recommendations for the future based on a systematic review of existing studies. *PLoS Medicine*.

[31] Kirkham J. J., Gorst S., Altman D. G. (2015). COS-STAR: A reporting guideline for studies developing core outcome sets (protocol). *Trials*.

[32] Implementation of Traditional Medicine Strategy 2014-2023. https://www.who.int/activities/implementation-of-the-WHO-traditional-medicine-strategy-2014-202

